# Clinical Cases

**Published:** 1895-10

**Authors:** J. R. Haynes

**Affiliations:** Indianapolis, Ind.


### CLINICAL CASES.

J. R. HAYNES, M. D., INDIANAPOLIS, IND.

In December, 1892, was called to see Miss ——.

Objective—Was about twenty years of age, light complexion, light brown hair, blue eyes; would weigh about one hundred and twenty pounds. Face and limbs waxy and enemic, and colorless; tongue colorless and flabby; abdomen puffed and decidedly dropsical; feet and legs dropsical and an indentation would remain for sometime before being replaced.

Had been in the hands of numerous "regulars," who had dosed her for all that she was worth, and finally told her that she was utterly incurable, and that she could not possibly live more than a month, and that there was no use of her trying for assistance as it would be perfectly useless, as there was no help for her.

Subjective—Mind very gloomy and despondent (probably what she had been told by her former attendants had something to do with it). Would cry at the least attempt to sympathize with her; everything looked dark and blank before her; felt as if she had no friends and that no one cared for her, at other times ill-humored.

Sensations—Sensation of weakness; numb feeling in the limbs, with dull feeling in the occiput in the evening.

Head—Headache from the nape extending through to the forehead with a dull feeling all through the whole head.

Head, outer—Falling out of the hair, which was dry and brittle, lifeless; would mat easily.

Eyes—Pressure as if behind the eyeballs; puffed condition of both upper and lower eyelids; disturbed vision, could not read but for a moment at a time, as everything would become all blurred and dance about.

Face—Pale, bloodless, and puffed.

Tongue—Pale and flabby, fissure in the centre, and bloodless.

Mouth—A collection of viscid mucus in the mouth with foul taste.

Throat—Accumulation of viscid sticky mucus in the throat and fauces, which was hard to clear out in the morning.

Desires—Craves large quantities of cold drinks, wants something that would be refreshing.

Eating—Very poor appetite, nothing suits her taste.

Stomach—Heavy feeling in the stomach, sensation of emptiness in the stomach.

Abdomen—Feeling of tension in the abdomen, with soreness to touch.

Stool—Tardy, infrequent, hard, dry stool, with itching about the anus.

Urine—Scanty, dark, yellowish, some smarting when passing it, strongly alkaline and heavily charged with albumen and with considerable fœtor; decomposed easily.

Sexual organs—Had not menstruated for nearly two years; acrid leucorrhœa, which caused considerable external irritation.

Breathing—Oppression of the chest, more especially in the upper portion when laying down; must have her head bolstered up high.

Heart—Sore pain in the region of the heart; pulse small and wavy.

Limbs, upper—Weakness and numbness and trembling of the hands; rapid growth of the finger-nails; pale and bloodless.

Limbs, lower—Legs go to sleep easily, tops of the feet œdematous and dropsical, which extended to near the knees, and doughy.

Exertion—Complete exhaustion upon the least exercise.

Fever—Sourish, unpleasant smelling sweat after slight exercise.

Skin—Pale and dropsical, easily dented.

Sleep—Nervous, restless sleep, dreaming of the most horrible fantasies as soon as she shut her eyes; dreams of seeing frightful hobgoblins, awakes in a fright, feels exhausted in the morning, has no ambition or life, sees nothing to live for.

Upon studying over this case I gave her Fluoric-acid$^{cm}$, one dose with Sac-lac. in water, to be taken one teaspoonful every two hours for one week, when the same dose was repeated with the Sac-lac. Very soon some slight improvement set in, and after four weeks she was kept on Sac-lac. The albumen began to disappear, the urine became acid, and menstruation made its appearance in about six weeks, and has continued regular since. In about three months the case became stationary, when two doses of Fluoric-acid were given one week apart, followed by Sac-lac. The Fluoric-acid was all the remedy that she had, and in about six months she was discharged as cured.

There has been no trouble up to this time, and I think that we may pronounce her case cured.

---

